# Combinational Treatment of Trichostatin A and Vitamin C Improves the Efficiency of Cloning Mice by Somatic Cell Nuclear Transfer

**DOI:** 10.3791/57036

**Published:** 2018-04-26

**Authors:** Rika Azuma, Kei Miyamoto, Mami Oikawa, Masayasu Yamada, Masayuki Anzai

**Affiliations:** ^1^Division of Biological Science, Graduate School of Biology-Oriented Science and Technology, Kindai University; ^2^Faculty of Biology-Oriented Science and Technology, Kindai University; ^3^Wellcome Trust/Cancer Research UK Gurdon Institute and Department of Zoology, University of Cambridge; ^4^Laboratory of Reproductive Biology, Graduate School of Agriculture, Kyoto University; ^5^Institute of Advanced Technology, Kindai University

**Keywords:** This Month in JoVE, Issue 134, Somatic Cell Nuclear Transfer, Histone Deacetylase Inhibitor, Trichostatin A, Histone H3 Lysine 9 Trimethylation, Vitamin C, Deionized Bovine Serum Albumin, Mouse, Hemagglutinating Virus of Japan Envelope

## Abstract

Somatic cell nuclear transfer (SCNT) provides a unique opportunity to directly produce a cloned animal from a donor cell, and it requires the use of skillful techniques. Additionally, the efficiencies of cloning have remained low since the successful production of cloned animals, especially mice. There have been many attempts to improve the cloning efficiency, and trichostatin A (TSA), a histone deacetylase inhibitor, has been widely used to enhance the efficiency of cloning. Here, we report a dramatically improved cloning method in mice. This somatic cell nuclear transfer method involves usage of Hemagglutinating virus of Japan Envelope (HVJ-E), which enables easy manipulation. Moreover, the treatment using two small molecules, TSA and vitamin C (VC), with deionized bovine serum albumin (dBSA), is highly effective for embryonic development. This approach requires neither additional injection nor genetic manipulation, and thus presents a simple, suitable method for practical use. This method could become a technically feasible approach for researchers to produce genetically modified animals from cultured cells. Furthermore, it might be a useful way for the rescue of endangered animals via cloning.

**Figure:**
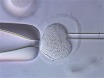


## Introduction

The SCNT technology enables the production of cloned animals by using only one somatic cell or a nucleus to be transferred to an enucleated oocyte. One of the purposes of the SCNT technique is the derivation of nuclear transfer embryonic stem cells (NT-ESCs) lines from cloned embryos. In 1998, Wakayama *et al*., reported producing a successfully cloned mouse named Cumulina for the first time^1^. Since then, the cloning of mice has been widely studied, and many important insights into nuclear reprogramming of somatic nuclei have been obtained. On the other hand, this technique is accompanied by numerous micromanipulation steps which are quite difficult to master, requiring intensive training of more than 3 months^2^.

Production of cloned mice using SCNT has evolved from the original Honolulu method^1^, the electrofusion method^3^, to the cell fusion method by Hemagglutinating virus of Japan (HVJ)^4^. However, the direct injection of a cell nucleus through the cytomembrane tends to detrimentally affect oocyte survival. The electrofusion is low in efficiency, since each cell membrane has different hardness, making it difficult to determine an optimal condition. The handling of HVJ is laborious because it requires specific equipment for the safety of researchers and laboratory animals. Recently, to fuse the donor cell and oocyte cytoplasm, HVJ-E has been used^5^. HVJ-E only has the ability to fuse membranes without the proliferative or infectious ability of viruses. Genomic RNAs of HVJ are completely inactivated in HVJ-E. The usage of HVJ-E thus supports easy handling of cell fusion during SCNT.

Several reports have shown that the treatment of SCNT embryos with TSA, a histone deacetylase inhibitor, significantly improves the production efficiency of live pups from less than 1% to 6.5%^6^,^7^. TSA treatment accelerates reprogramming through modifying histone marks in SCNT embryos^8^. Recently, injection of particular mRNAs, the histone lysine demethylase subfamily 4 (KDM4), which remove histone H3 lysin 9 (H3K9) trimethylation in SCNT embryos, especially at reprograming-resistant regions, has been shown to increase development of cloned mouse embryos^9^. Meanwhile, VC, which also serves as a histone modifier, has decreased trimethylation of H3K9^10^. Furthermore, VC enhances embryonic development in porcine SCNT^10^. It has been reported that the injection of dBSA into SCNT embryos leads to the improvement of embryonic development^11^.

We previously found that the combination of small molecules, namely TSA and VC, together with dBSA, dramatically enhanced development of SCNT embryos^12^. Here, we detail the previously reported SCNT method for mice, which represents highly efficient and simple cloning procedures^12^. We also describe the handling of HVJ-E. These could help many researchers in the field of developmental and reproductive biology to preserve genetic resources or produce genetically modified animals through this SCNT method.

## Protocol

All animal procedures conformed to the guidelines of Kindai University for the Care and Use of Laboratory Animals.

### 1. Preparation of Culture Media

1. Prepare the modified KSOM (mKSOM) medium for embryo culture, consisting of 95 mM NaCl, 2.5 mM KCl, 0.35 mM KH_2_PO_4_, 0.2 mM MgSO_4_ · 7H_2_O, 1.71 mM CaCl_2_ · 2H_2_O, 0.2 mM D(+)-Glucose, 0.2 mM Sodium Pyruvate, 1.0 mM L-Glutamine, 1.0 g/L Polyvinylpyrrolidone (PVP), 25.07 mM NaHCO_3_, 10 mM Sodium DL-Lactate, 0.05 g/L Penicillin, and 0.05 g/L Streptomycin in sterile water.
2. Prepare the Hepes-buffered CZB (HCZB) medium for embryo manipulation, consisting of 81.5 mM NaCl, 4.8 mM KCl, 1.7 mM CaCl_2_ · 2H_2_O, 1.18 mM MgSO_4_ · 7H_2_O, 1.18 mM KH_2_PO_4_, 0.11 mM EDTA · 2Na, 36.1 mM Sodium DL-Lactate, 5.55 mM D(+)-Glucose, 0.025 g/mL Penicillin, 0.035 g/L Streptomycin, 0.014 mM Phenol Red, 1.0 g/L Polyvinyl alcohol, 5.0 mM NaHCO_3_, and 20.0 mM Hepes sodium salt in sterile water.
3. Prepare the activation medium for oocyte activation: mKSOM medium supplemented with 50 nM TSA, 2 mM EGTA, 5 µg/mL cytochalasin B (CB), and 5 mM SrCl_2_.

### 2. Preparation of Deionized Bovine Serum Albumin (dBSA)

1. Dissolve 1.2 g of bovine serum albumin (BSA) in 10 mL of sterile water at room temperature (a final concentration of 12%).
2. Add approximately 0.12 g of mixed ion-exchange resin beads to the above prepared 12% of BSA in sterile water at room temperature with gentle stirring.
3. When the beads change color from blue-green to gold, recover the supernatant solution using a pipette. Then replace the beads with the fresh ones (Figure 1). NOTE: The replacement of beads is normally performed only once. It possibly requires a few replacements to complete deionization. Only as a guide, if the color of beads remains unchanged from blue-green to gold, it indicates that ion exchange is complete.
4. Recover the solution after confirming that there is no color change in the beads. NOTE: If the recovered solution becomes cloudy, centrifuge it to prevent clogging the filter (175 x g, 5 min).
5. Supplement the recovered the solution with 250 µL of 10.5% NaHCO_3_. NOTE: This process is intended for the dBSA solution to neutralize pH condition. However, NaHCO_3_ can be dispensable.
6. Sterilize the supernatant using a 0.45-µm filter, and store at -20 °C as dBSA stock solution.

### 3. Oocyte Collection

NOTE: All mice were maintained in light-controlled and air-conditioned rooms.

1. Super-ovulate each female B6D2F1 mouse (aged 8 - 10 weeks) by intraperitoneal injection of 7.5 IU of pregnant mare serum gonadotropin (PMSG) and 7.5 IU of human chorionic gonadotropin (hCG) 48 h after the PMSG injection.
2. Perform euthanasia by cervical dislocation 14 - 16 h after hCG injection. Incise the abdomen to access the reproductive tract using standard dissection techniques^13^. Briefly, pinch the skin and make a small lateral incision at the midline with scissors. Hold the skin firmly above and below the incision and pull the skin toward the head and tail. Cut the peritoneum using scissors, push the coils of the gut out of the way and confirm the presence of the two horns of the uterus, the oviducts, and the ovaries.
3. Extirpate oviducts with small straight scissors and tweezers, place on a sheet of filter paper to wipe the blood off of the surface. Set the oviducts in mineral oil. Then, cut up the ampulla of the oviduct using dissection needles, and move the cumulus-oocyte complexes coming from the ampulla of the oviduct into 200 µL drops of HCZB medium with 0.1% hyaluronidase. Incubate for 5 min on a warming plate. Caution: An exposure of longer than 10 min in HCZB medium with 0.1% hyaluronidase would be detrimental for the oocytes.
4. Confirm that the cumulus cells are released from the cumulus-oocyte complexes under a stereomicroscope, transfer second meiotic metaphase (MII) stage oocytes with some remaining cumulus cells to mKSOM medium containing 0.3% dBSA. Then, wash the MII stage oocytes four times by pipetting up and down (using a pipette of <100 µm inner diameter) in mKSOM medium containing 0.3% dBSA for detaching the remaining cumulus cells. NOTE: The use of a small pipette facilitates detachment of the cumulus cells.
5. Incubate Mll stage oocytes in mKSOM medium containing 0.3% dBSA at 37 °C under 5% CO_2_ incubator until enucleation. NOTE: A small pipette with a diameter that is slightly larger than that of the oocyte is recommended.

### 4. Preparation of Donor Cells for Nuclear Transfer

1. After steps 3.3 - 3.5, denuded oocytes are isolated from the cumulus-oocyte complexes. Transfer a small amount (approximately 2 µL) of the remaining cumulus cells dispersed from the cumulus-oocyte complexes in HCZB medium with 0.1% hyaluronidase to HCZB medium with 6% dBSA by using a pipette under a stereomicroscope.
2. Place the cumulus cells in HCZB medium with 6% dBSA on the warming plate at 37 °C until needed for SCNT. NOTE: When fibroblast cells (*e.g*., murine fetal fibroblast cells) are used as donor cells, detach the cells from a tissue culture dish and centrifuge them into a pellet. Resuspend the pellet of cells in HCZB medium containing 6% dBSA.

### 5. Enucleation of Oocytes

1. Prepare 9% PVP medium by adding 0.9 g PVP to 10 mL HCZB medium, keep in the refrigerator overnight (4 °C), and then sterilize it using a 0.45 µm filter.
2. Prepare CB stock solutions (concentration of 1 mg/mL) by adding 5 mg of CB to 5 mL of dimethyl sulfoxide (DMSO). Dilute the CB stock solution with HCZB medium (a final concentration of 5 µg/mL) and use as enucleation solution.
3. Wash the Mll stage oocytes in 20 µL of HCZB medium containing 5 µg/mL CB (enucleation solution) by inhaling and exhaling through the sterilized pipette (inside diameter: 150 µm to 200 µm). Repeat the washing procedure five times. Wait approximately 10 min on the warming plate in enucleation solution before starting the enucleation process.
4. Prepare the enucleation chamber as shown in Figure 2A. Transfer the Mll oocytes to the enucleation chamber by inhaling and exhaling using the pipette (inside diameter: 150 µm to 200 µm); place 4 µL of enucleation solution and cover the chamber with mineral oil. NOTE: Instead of a chamber, a cover of a 60 mm Petri dish can be used.
5. Identify the spindles and chromosomes of the MII stage oocyte under a microscope (400X) at room temperature. Orient the Mll stage oocyte with the pronounced first polar body, so that the positioning of the spindle and chromosome is at the 3 or 9 o'clock (Figure 2B) position using a holding pipette and micropipette. NOTE: The microscope used has lenses of 400X magnification in total. Also, the objective lens N.A is 5X/0.12 and 40X/0.55. The piezo pulse driving of the pipette permits rapid drilling of the zona pellucida. In addition, a laser system (*e.g.*, XYClone) is also usable for the drilling of the zona pellucida in place of the piezo driving system.
6. Set the piezo pulse intensity to 3 - 6, and drill to the zona pellucida using a micromanipulation pipette (7 - 8 µm inner diameter flat-ended tip) with the piezo pulse driving. After opening a hole in zona pellucida, completely enucleate the spindles and chromosomes with a minimal amount of cytoplasm (Figure 2C). NOTE: During this process, oocytes tend to tightly attach to the dish bottom or pipette due to the medium without BSA^14^. Within 10 min, the enucleation process must be completed, and the enucleated oocytes need to be returned to the incubator. The appropriate number of oocytes that can be manipulated in one enucleation is between 10 and 20. For larger batches of oocytes, the enucleation procedure is repeated.
7. Confirm the absence of chromosomes in cytoplasm under visible light (Figure 2C, middle), then wash the enucleated oocytes thoroughly in mKSOM medium containing 0.3% dBSA lacking CB. Introduce the dish to the incubator at 37 °C under 5% CO_2_ until cell fusion. NOTE: After enucleation, the cytoplasm removed from ooplasm contains the spindles and chromosomes.

### 6. Fusion of a Donor Cell and an Enucleated Oocyte

1. **Preparation of HVJ-E**
  1. Add 260 µL of ice-cold HVJ-E suspension solution to lyophilized HVJ-E (from the kit, see the **Table of Materials**) and pipette up and down until fully suspended. NOTE: The appropriate amount of lyophilized HVJ-E is prepared in a bottle, and 260 µL HVJ-E suspension solution is included in the kit.
  2. Prepare 5 µL aliquots of the HVJ-E solution, and then store at -80 °C until cell fusion. Caution: Carefully treat the HVJ-E on ice, since it is temperature-sensitive. After completion of the experiment, appropriately autoclave HVJ-E materials and containers to completely inactivate virus components.
2. **Cell Fusion**
  1. Dilute the 5 µL of HVJ-E solution with 20 µL of the cell fusion buffer immediately before use. Place the diluted HVJ-E solution on ice until use. NOTE: The cell fusion buffer is included in the HVJ-E kit.
  2. Prepare the cell fusion chamber as shown in Figure 3A. Transfer the enucleated oocytes to HCZB medium on the cell fusion chamber using the pipette (inside diameter: 150 µm to 200 µm); place 4 µL of each solution (HCZB medium containing 6% -BSA for donor cells, HVJ-E solution,HCZB medium,9% PVP in HCZB medium) and cover the chamber with approximately 1 mL of mineral oil. NOTE: The appropriate number of enucleated oocytes that are transferred to the chamber for manipulation during one cell fusion procedure is from 5 to 30. For larger batches of oocytes, the cell fusion procedure is repeated. Instead of chamber, a 60-mm Petri dish cover can be used, if available.
  3. Place the enucleated oocytes in HCZB medium under a microscope at 400X magnificationat room temperature. Aspirate the donor cells using the micromanipulation pipette (6-7 µm inner diameter flat-ended tip), and expel cells into HVJ-E suspension solution.
  4. Then aspirate cumulus cells one by one with the HVJ-E suspension solution to be equally separated from each other, enabling serial cell fusion. Keep the enucleated oocytes in HCZB medium using a holding pipette. Drill the zona pellucida with the micromanipulation pipette with the piezo pulse (Intensity of 3 - 6, Speed of 2 - 3).
  5. After opening the hole in the zona pellucida, place a cumulus cell tightly to the oocyte membrane, along with HVJ-E suspension solution with a volume 5 times the volume of a cumulus cell, without going through the oocyte membrane. NOTE: Prepare 6 - 7 µm inner diameter flat-ended tips of micromanipulation pipettes for cumulus cells. Pipettes must be washed regularly using 9% PVP medium for maintaining smooth cell release. Steps 6.2.3 - 6.2.5 must be finished within 10 min, or the efficiency of cell fusion will be lower. Membrane fusion activity of HVJ-E is inactivated by autoclaving, treatment with detergent, or 70% ethanol. After the experiment, the HVJ-E solution must be appropriately disposed of.
  6. After manipulation of cell fusion, promptly transfer the oocytes to mKSOM medium containing 0.3% dBSA, and move to an incubator at 37 °C under 5% CO_2_ for 1 h.

### 7. Activation of the Reconstructed Oocytes and Treatment with Trichostatin A and Vitamin C

1. **Preparation of TSA**
  1. Dispense 5 mM TSA solution into 3 µL aliquots, and store the solution at -20 °C.
  2. Add 2.5 µL of 5 mM TSA stock solution to 1 mL of DMSO (a concentration of 12.5 µM).
  3. Dilute 8 µL of 12.5 µM TSA stock solution in 2 mL of the activation medium and mKSOM medium (a final concentration of 50 nM).
2. **Preparation of VC**
  1. Add 1 mg of VC into 1 mL of sterile endotoxin-free water, and store at -20 °C.
  2. Add the VC stock solution to mKSOM medium (a final concentration of 10 µg/mL).
3. **Activation of the Reconstructed Oocytes**
  1. One hour after cell fusion, check the premature chromosome condensation (PCC) in the reconstructed oocytes (Figure 3B) using a microscope (400X).
  2. Transfer the reconstructed oocytes to the activation medium (see the step 1.3) containing TSA, and incubate for 6 h at 37 °C under 5% CO_2_ in air (Figure 3C). Observe the formation of pro-nuclei in the SCNT embryos, then apply 2 more hours of TSA treatment in mKSOM medium containing 0.3% dBSA (Figure 2C).
4. Transfer the TSA-treated embryos to mKSOM medium supplemented with VC and incubate for 7 h, as shown in Figure 3D.
5. After 7 h of the VC treatment, transfer the VC-treated embryos to mKSOM medium containing 0.3% dBSA, and incubate for 4 days at 37 °C under 5% CO_2_ in air. NOTE: To produce cloned mice, transfer morphologically normal 2-cell stage embryos into the oviducts of pseudo-pregnant female mice (MCH(ICR)) on the day when a vaginal plug is found (Day 0.5 of pseudopregnancy)^15^. After 19.5 days, the number of implantation sites and newborns are recorded after cesarean section.

## Representative Results

To produce cloned mouse embryos, cumulus cells and fetal fibroblast cells were used. The number of reconstructed oocytes and development to the 2-cell stage after oocyte activation are shown in **Table 1**. A very high rate of pronuclear formation (89 to 100%) and development to the 2-cell stage (77 to 89%) were observed under all conditions. Some of cloned embryos, which were derived from the cumulus cells and developed to the 2-cell stage, were transferred to oviducts of pseudo-pregnant females. Six cloned offspring out of 72 transferred embryos were produced from three pregnant females by the serial treatment of TSA and VC (Figure 4). Approximately 15% of cloned embryos have been reported to develop to term by following this SCNT procedures^12^. In addition, the transfer of cloned 2-cell embryos at other institutions has achieved 9 to 15% live offspring, which represents better development than the single TSA treatment on cloned embryos. Moreover, the treatment with TSA and VC significantly improved the efficiency of *in vitro* embryonic development to the blastocyst stage (**Table 2**, *P *<0.05, Student's t-test). These *in vitro* developmental data demonstrate that the positive effect of TSA and VC is limited neither by mouse strains nor by cell types. These results suggest that this SCNT method facilitates developmental ability of the cloned embryos.

**Figure:**
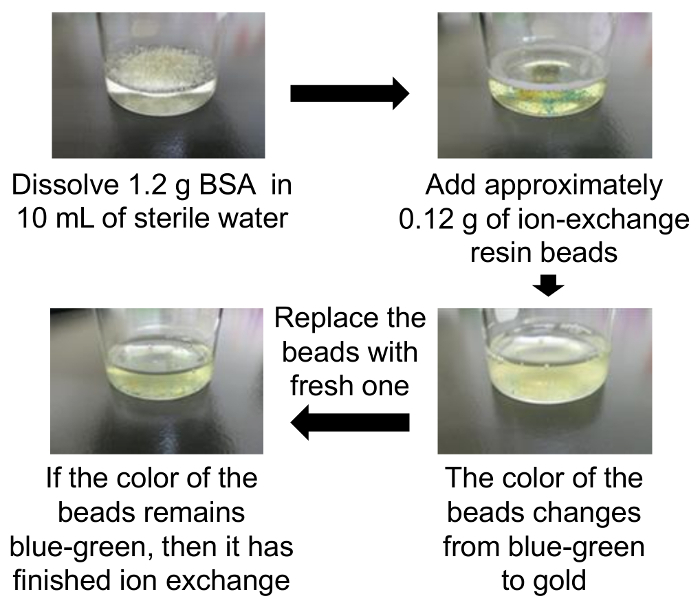

**Figure 1: Preparation of dBSA.** Step-by-step procedures for preparing dBSA solution are depicted. The extent of ion exchange can be judged by the color change of the beads. The upper left figure shows that 1.2 g of BSA is dissolved in 10 mL of sterile water at room temperature. After BSA is dissolved, the ion-exchange resin beads are added (upper right). When the mixture of BSA solution with ion-exchange resin beads change color from blue-green to gold (bottom right), replace the beads with fresh ones. The bottom-left figure shows that the color of beads remains blue-green, and ion exchange is finished. Please click here to view a larger version of this figure.

**Figure:**
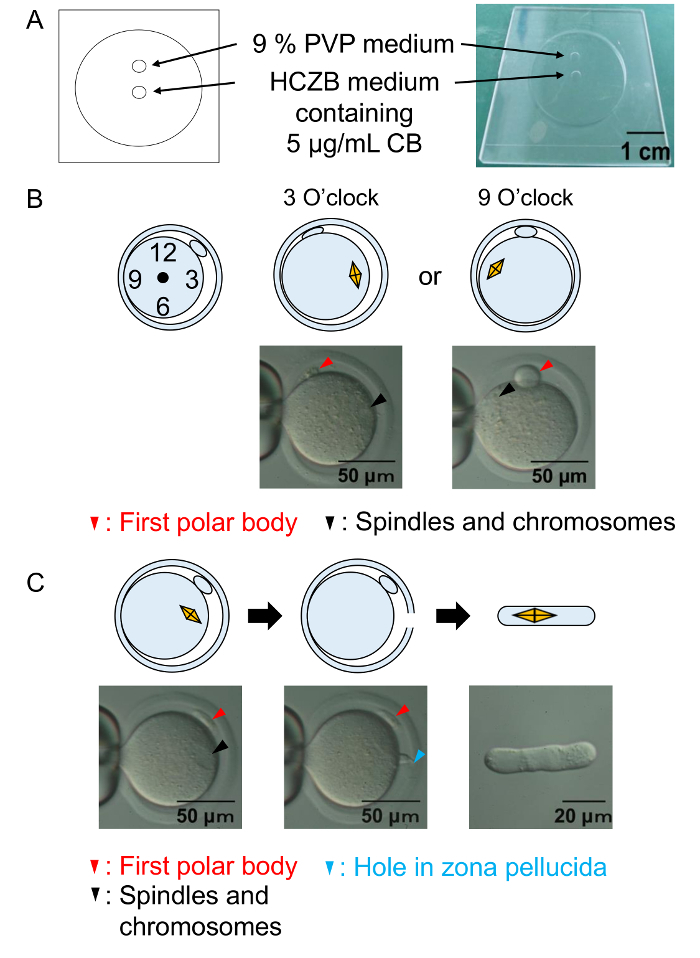

**Figure 2: Enucleation procedures.** (**A**) An illustration of the enucleation chamber. Enucleation of oocytes is performed in the HCZB medium with CB. For the piezo driving system, a spot of 9% PVP medium is used to prepare the glass pipette for enucleation. Spots are covered by mineral oil. (**B**) A diagram and a micrograph show the position of the spindles and chromosomes before enucleation. Black arrowheads: the spindles and chromosomes. Red arrowheads: the first polar body. (**C**) A diagram and a micrograph to show successful enucleation. Black arrowheads: the spindles and chromosomes. Red arrowhead: the first polar body. Blue arrowhead: the hole in zona pellucida. Please click here to view a larger version of this figure.

**Figure:**
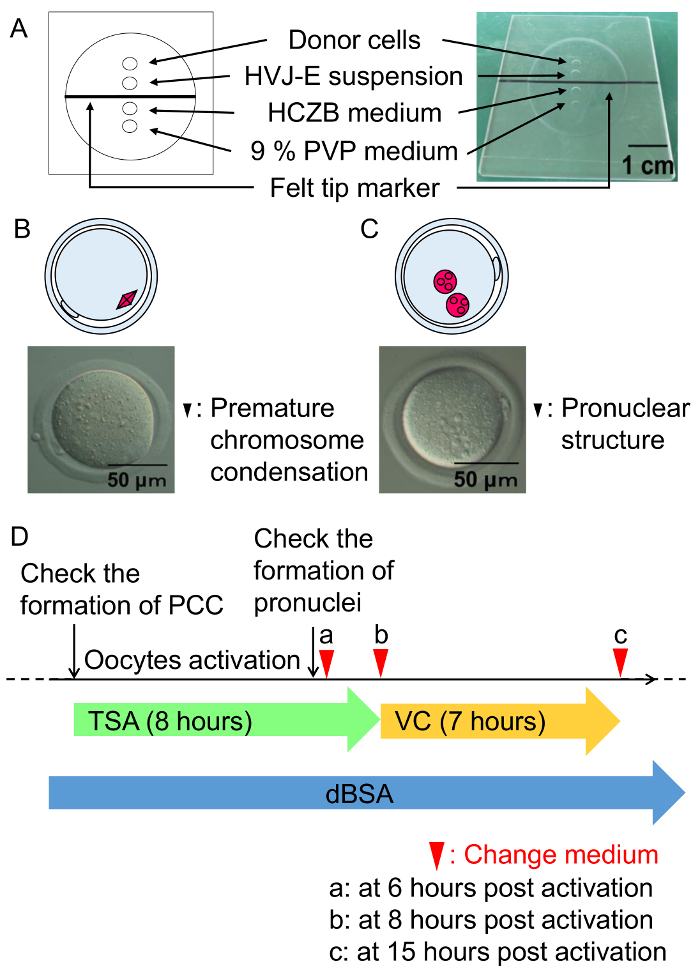

**Figure 3: Cell fusion procedures and culture condition of SCNT embryos.** (**A**) An illustration of the cell fusion chamber. Cell fusion is performed in the HCZB medium containing 6% dBSA. A spot of 9% PVP medium is used to prepare the glass pipette for cell fusion. Spots are covered by mineral oil. (**B**) A diagram and a micrograph of the premature chromosome condensation formed one hour after cell fusion (black arrowhead). (**C**) A diagram and a micrograph of the pronuclear structure formed six hours after activation (black arrowheads). (**D**) Scheme of the TSA, VC, and dBSA treatment for SCNT embryos. Green arrow represents treatment with TSA, followed by incubation with VC (yellow arrow) under mKSOM medium containing with 0.3% dBSA (blue arrow). Arrow heads indicate the timing for changing medium. Please click here to view a larger version of this figure.

**Figure:**
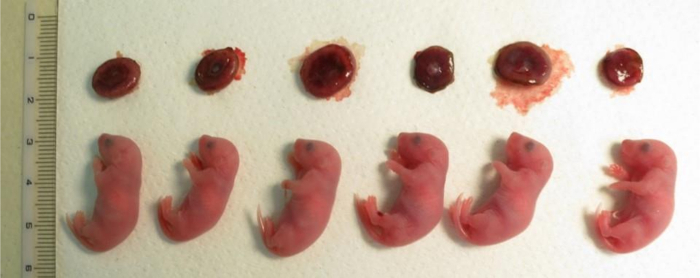

**Figure 4: Cloned offspring derived from cumulus cells just after caesarean section after 19.5 days of pregnancy.** There are placentae at the top row. The cloned offspring shown here were generated in one nuclear transfer experiment from three foster mothers. The placenta size was 1.5 to 2 times bigger than the size of those produced by *in vitro* fertilization. Please click here to view a larger version of this figure.

**Table:** 

**Group**	**Donor cell type**	**Mouse strain**	**No. of oocytes used**	**No. of oocytes fused**	**No. of oocytes showing premature chromosome condensation**	**No. of oocytes showing pronuclei formation (%)**	**No. of pronuclei-formed oocytes that developed to 2-cell embryos (%)**
TSA, VC	cumulus	C57BL/6× DBA/2	84	82	82	81 (99)	72 (89)
untreated	cumulus	C57BL/6× DBA/2	84	82	82	82 (100)	68 (83)
TSA, VC	fetal fibroblast	MCH(ICR)×MCH(ICR)	202	171	169	151 (89)	124 (82)
untreated	fetal fibroblast	MCH(ICR)×MCH(ICR)	201	171	147	142 (97)	109 (77)

**Table 1: Effects of the TSA and VC treatment on the** ***in vitro*** **development of cloned mouse embryos to the 2-cell stage.**

**Table:** 

**Group**	**Donor cell type**	**Mouse strain**	**No. of 2-cell embryos used**	**No. of 2-cell embryos developed to each stages (%)**
**4-cell**	**morula**	**blastocyst**
TSA, VC	cumulus	C57BL/6× DBA/2	152	152 (100)	149 (98)	135 (89) ^a^
untreated	cumulus	C57BL/6× DBA/2	83	47 (57)	41 (49)	32 (39) ^b^
TSA, VC	fetal fibroblast	MCH(ICR)×MCH(ICR)	124	110 (89)	101 (81)	88 (71) ^c^
untreated	fetal fibroblast	MCH(ICR)×MCH(ICR)	109	54 (50)	45 (41)	29 (27) ^d^
^a-b, c-d ^Different superscripts within the same donor cells represent significant differences (P < 0.05)		

**Table 2: Effects of the TSA and VC treatment on the** ***in vitro*** **development of cloned mouse embryos to the blastocyst stage.**

## Discussion

In conclusion, these results suggest that the presented SCNT method could reduce technical difficulties, and increase the efficiency of SCNT without requiring genetic modifications and mRNA supplementation (**Table 1**, **Table 2**), and ensure stable production of cloned embryos. This method enables us to reconstruct more SCNT embryos than conventional methods due to the better survival rate and simplified protocol. In this protocol, one critical step is cell fusion. To successfully produce cloned mice, it is vital to ensure that the proper amount of HVJ-E described in the protocol is maintained during the cell fusion process and oocytes need to be returned to the incubator within 10 min during the steps 6.2.3 - 6.2.5. Since 20 to 30 donor cells can be aspirated together with HVJ-E at a time in the manipulation pipette, the number of oocytes obtained by one operation is larger than that of the existing method. In the end, we can produce about 20 to 30 re-constructed oocytes within 10 min. Even when working with a large batch of oocytes (100 or more), the cell fusion procedure should take an hour or less by repeating the steps 6.2.3 - 6.2.5. The method and techniques presented here can serve as efficient protocols with simplified technical requirements.

At present, the molecular mechanisms underlying the development of SCNT embryos are still unclear. This improved SCNT method also contributes to studying such reprogramming mechanisms, since this method can produce many cloned embryos in only one experiment. This method uses somatic cells with intact cell membranes for cell fusion. Thus, it may be possible to apply this approach to other cells such as tail tip cells^16^, sertoli cells^17^, and embryonic stem (ES) cells^18^. When conventional SCNT methods are used for injecting relatively large cells, such as tail tip cells, directly into the oocyte cytoplasm, it becomes even more technically demanding to obtain live embryos. In addition, relatively hard cells, such as sertoli cells, are difficult to break by pipetting for injecting. When these various cell types are considered, the cell fusion method utilizing HVJ-E is simple and effective. Although the value and safety of HVJ-E have been convincingly demonstrated^19^,^20^, it might be important to re-consider the feasibility of using HVJ-E for producing cloned animals for agricultural or biomedical purposes.

Moreover, recently one group successfully produced cloned mice derived from urine cells^21^. To rescue endangered mammalian species, henceforth SCNT using the cells collected in a non-invasive manner, such as the urine cell, will be ideal. More recently, another group has directly generated cloned mice using antigen-specific CD4^+^ T cells^22^. It would be interesting to examine if this method is also applicable to efficiently clone mice from such cells. Furthermore, Latrunculin A has been reported as a better alternative for inhibiting actin polymerization during enucleation and parthenogenetic activation of SCNT oocytes^23^. Future study may reveal whether the Latrunculin A treatment, instead of cytochalasin B, further improves generation of cloned offspring. Additionally, the TSA treatment has been successfully used in mice, pigs^24^, and rabbits^25^ by changing treatment time, period, and concentration. Furthermore, VC not only enhances embryonic development in porcine SCNT^10^, but also improves iPS cell production in humans and mice^26^. Thus, it is plausible to speculate that TSA and VC treatment can also be applied to other mammalian species, and we may need to optimize the treatment time of TSA and VC for each species.

In conclusion, this method would make it possible to generate cloned mice with a practical level of efficiency with simple procedures. Therefore, the results of this study could lead us to use the SCNT technology for preserving the genetic resources of rare animals, and for understanding the molecular mechanisms of nuclear reprogramming and early embryonic development.

## Disclosures

The authors have nothing to disclose.
